# How the Interplay Between the Commensal Microbiota, Gut Barrier Integrity, and Mucosal Immunity Regulates Brain Autoimmunity

**DOI:** 10.3389/fimmu.2019.01937

**Published:** 2019-08-16

**Authors:** Martina Antonini, Marta Lo Conte, Chiara Sorini, Marika Falcone

**Affiliations:** ^1^Experimental Diabetes Unit, Division of Immunology, Transplantation and Infectious Diseases, IRCCS San Raffaele Scientific Institute, Milan, Italy; ^2^Immunology and Allergy Unit, Department of Medicine, Solna, Karolinska Institute and University Hospital, Stockholm, Sweden

**Keywords:** autoimminity, microbiota, T cells, gut barrier, central nervous system

## Abstract

The intestinal barrier provides the host with a strong defense line against the external environment playing also a pivotal role in the crosstalk between the gut microbiota and the immune system. Notably, increasing lines of evidence concerning autoimmune disorders such as Multiple Sclerosis (MS) report an imbalance in both intestinal microbiota composition and mucosal immunity activation, along with an alteration of gut barrier permeability, suggesting this complex network plays a crucial role in modulating the course of autoimmune responses occurring in tissues outside the gut such as the central nervous system (CNS). Here, we review current knowledge on how gut inflammation and breakage of gut barrier integrity modulates the interplay between the commensal gut microbiota and the immune system and its role in shaping brain immunity.

## Introduction

The human gastrointestinal tract is equipped with physical and biological barriers whose function is not only to isolate the internal host's milieu from the external environment but also to regulate the immune system, absorption of nutrients, and to limit the access of microorganisms, both commensal, and pathogens. Hence, the intestinal mucosa operates in a dynamic manner in order to maintain intestinal integrity and immune homeostasis. Alterations of gut barrier integrity have been associated not only to inflammatory bowel diseases (IBD) but also to autoimmune disorders occurring outside the gut, such as Multiple Sclerosis (MS) both in experimental models and humans. These alterations are associated with dysbiosis, i.e., modification of microbiota composition, along with a persistent activation of the immune system within the gut mucosa (1). The mechanisms through which gut dysbiosis, breakage of gut barriers, and brain autoimmunity are linked are still unknown, but it has been proposed that a condition of dysbiosis can promote inflammation and morphological and functional changes of intestinal mucosa thus favoring uncontrolled passage of macromolecules, microorganisms or their derivates from the intestine to the systemic circulation where they activate myelin-reactive T cells. Here we will review the current knowledge on how the commensal microbiota and the gut barriers modulate the immune system within the gut mucosa and systemically and how this could influence the pathogenesis of MS (2, 3).

## The Crosstalk Between the Commensal Microbiota and the Immune System

More and more revalued and increasingly considered by the recent scientific bibliography is the concept of intestinal ecosystem, by which functions and interactions among mucosal barrier, local immune system, and intestinal microflora are defined. Inside the gut microbial community, the esteemed number of species changes, but it is generally accepted that the human microbial compartment includes at least 10^14^ microbial cells, overall 10 times more than the total human somatic cells (4). The advances of molecular biology techniques, including ribosomal RNA 16S sequencing and metagenomics, have been instrumental in identifying the large biodiversity of the mammalian intestinal microbiota. In fact, microbiota organization is extremely diverse and influenced by many environmental and time factors. In the mammalian microbiota are five predominant *phyla* present (Bacteroidetes, Firmicutes, Actinobacteria, Proteobacteria e Verrucomicrobia). Firmicutes include several genera such as *Clostridium, Lactobacillus* and *Ruminococcus*, as well as *Eubacterium, Fecalibacterium*, and *Roseburia*; Bacteroidetes include *Bacteroides, Prevotella* and *Xylanibacter* genera; Proteobacteria involve *Escherichia* and *Desulfovibrio*, while *Akkermansia* genus belong to Verrucomicrobia phylum (5). Although every mammalian intestine harbors a unique microbiota profile, there is a balance in its composition conferring benefits toward the host, whose alteration negatively affects the overall health status (6). Indeed, studies of germ-free (GF) or antibiotic-treated mice demonstrated that functions of commensal bacteria are important in several mechanisms, including, but not restricted to, the maturation of the immune system, and other physiological processes like resistance to pathogenic infections, nutrient absorption and maintenance of intestinal barrier functions (7, 8). All these functions are closely dependent on the heterogeneous and dynamic features of the surrounding environment and they are also fundamental in maintaining the homeostasis in the intestinal environment.

Over the past few years, broad evidence of a pivotal role of the microbiota in shaping the immune responses has been collected. It has long been acknowledged that, when compared to wild-type, germ free mice exhibit imbalanced immune development characterized by immature gut-associated lymphoid tissues (GALT), decreased numbers of intestinal lymphocytes and decreased levels of both antimicrobial peptides and immunoglobulin A, two components that are essential to contrast enteric infections (9). On the other hand, the intestinal immune system is crucial to discriminate between invasive or opportunistic organisms and harmless ones, i.e., the commensal microbiota, adopting an immunological tolerance toward the latter. Hence, intestinal microbiota regulates the development and function of the immune system which in turn, shape the microbial community and regulates immune responses against the bacterial species on mucosal surfaces.

Specific resident bacteria play an important role in shaping the immunological features of different immune cell subsets. Hence, knowledge of mechanisms through which the gut microbiota regulate T cells differentiation is highly relevant in the field of immunology and autoimmune disease.

For example, it has been observed that GF mice show reduced number of Th1 and Th17 cells, as well as a reduction of IL-17 and IL-22 cytokines and overall, a decrease of Lamina Propria (LP) associated -CD4^+^ lymphocytes (10–12). Th17 cells, in particular, normally play a key role in host defense from pathogens, and several studies also describe their involvement in the pathogenesis of autoimmune diseases (13). A decrease in this cell subset has been correlated to the absence or reduction of segmented filamentous bacteria (SFB) in the human microbiota, suggesting their potent capacity in triggering Th17 cells differentiation and thereby, IL-17 and IL-22 induction (14, 15). Resident intestinal bacteria are also essential for the induction and the development of Foxp3^+^ cells in the gut. In the absence of commensals, the amount of Foxp3^+^ Helios^−^ T_reg_ (intestinal T_reg_) is significantly reduced at colonic lamina propria level (16). However, by re-balancing the intestinal microbiota with certain bacterial species, intestinal T_reg_ subset can be restored (16–18). Indeed, colonization of GF mice with *Clostridium* strains triggered the transforming growth factor β (TGF-β) expression by intestinal epithelial cells, and thereby, promoting the differentiation of Foxp3^+^ T regulatory lymphocytes in the colonic lamina propria (18). Furthermore, in a mouse model of experimental colitis, through a polysaccharide-A (PSA)- mediated mechanism, a molecule univocally expressed on its surface, the commensal *Bacteroides fragilis* has been shown to be able to stimulate the development of T regulatory cells and to increase their suppressive capacity by inducing IL-10 anti-inflammatory cytokine (19). With similar mechanisms, the gut bacteria also have an important role in CD8^+^ T cell-mediated immune responses in the intestine. In fact, a reduced number of intestinal CD8^+^ T cells have been observed in GF mice, suggesting that signals from commensals are necessary in order to maintain the pool as well as the function of intestinal CD8^+^ T lymphocytes (20). Furthermore, gut microbiota also affects the development of intestinal-resident B lymphocytes residing at lamina propria level, given that GF mice have a reduced number of B cell in the intestinal LP (21). Such cell population is also responsible for the IgA production, which in turn, are strong regulators of microbiota composition. Hence, this has importance not only in order to reach a broad diversification for IgA at mucosal sites, but also to establish immune tolerance toward commensal microorganisms (22). Collectively, these studies demonstrate that our intestinal microbial community has a broad and long-lasting effect on the development and induction of T and B cell responses in the gastrointestinal tract.

Recent studies showed that is not the presence of a single or a consortium of bacterial strains that modify gut mucosal immunity but rather the functional metabolic profile that different bacterial species induce within the intestinal milieu. In fact, the gut microorganisms produce a broad range of metabolites through the anaerobic fermentation of exogenous and undigested dietary components as well as endogenous ones that are synthetized by both gut bacteria and the host. The interaction between microbial metabolic products and the host immune cells within the gut mucosa is fundamental to regulate differentiation of T cells with regulatory (T_reg_ cells) or effector (T_eff_ cells) properties. Among these metabolites there are, for instance, short-chain fatty acids (SCFAs), mainly acetate, propionate, and butyrate. Resulting all from dietary fiber fermentation, acetate and propionate are mainly produced by Bacteroidetes, while butyrate is produced by Firmicutes (23). SCFAs have been studied for a long time for their capacity to increase the number and function of regulatory T lymphocytes in the gut (24). In addition, it has been reported that these metabolites are involved in promoting gut barrier function and inducing anti-inflammatory effects through the inhibition of the transcription factor NF-κB, as well as in influencing gene transcription by counteracting histone-deacetylase (HDAC) activity (25–27). Another example of microbial metabolites that affect the intestinal immune system activity is provided by tryptophan derivatives metabolites that bind the aryl hydrocarbon receptor (AhR). Initially recognized for its involvement in xenobiotics metabolism, AhR was further investigated for its role in regulating mucosal immune responses (28). In such overview, tryptophan catabolism and production of indole-3-aldehyde by the commensal *Lactobacillus reuteri* has been shown to be able to inhibit the overgrowth of *Candida albicans* in mice gut by binding AhR and triggering the IL-22 pathway, thus protecting the mucosa from infection (29). In addition to tryptophan, intestinal bacteria are involved in the production of arginine derivatives such as polyamines, diamine, spermidine, and spermine, capable of exerting immune-modulatory effects, for instance, by enhancing the homeostasis of both intestinal mucosa and resident immune cells (30, 31). Hence, microbiota regulate immune responses in the gastrointestinal tract both directly and indirectly, by coordinating immune cell subsets to different conditions and regulating inflammatory responses. For these reasons, to elucidate molecular and metabolic pathways, regulatory metabolites as well as microorganisms involved in their production, is crucial to understand the pathogenesis of immune-mediated disorders.

## The Role of Physical and Biological Gut Barriers in Regulating the Crosstalk Between the Commensal Microbiota and the Immune System

The complex and dynamic interaction between the commensal microorganisms and the gut immune system is regulated at the gut barriers level through several mechanisms. Intestinal microorganisms and/or microbial metabolites may affect both immune system and epithelial barrier and, at the same time, intestinal epithelial cells (IECs) and the mucus layer are able to strongly influence immune responses and to shape the microbial composition. This complex network is fundamental to maintain immune homeostasis at the intestinal level.

Although gut commensals play a key role in orchestrating intestinal immune responses, an abnormal stimulation of mucosal immunity as well as a microbial systemic spreading, may lead to harmful intestinal inflammation and excessive immune activation. To avoid such an occurrence, intestinal microbes are compartmentalized in the lumen and remain isolated from intestinal immune system by physical and functional barriers working in a coordinated manner, consisting of the mucus layer, the intestinal epithelial barrier (IEB) and the gut vascular barrier (GVB) (8). In the colon, the mucus layer covering the epithelial one is organized in two zones: an inner dense layer, usually sterile and inhabited by immune system cells, and an outer layer colonized by commensal bacteria (32). The intestinal mucus is composed mainly by mucins which represent complex agglomerates of glycoproteins characterized by specific O-linked glycans produced by globet cells (33). The main function of the mucus layer is to limit the interaction between the gut mucosa and harmful molecules or pathogens present in the intestinal lumen and thus, to regulate the passage of food and microbial products into the gut tissue and systemic circulation. However, it is now clear that the mucus layer is also fundamental to both shaping the composition of gut microbial community and regulate the interaction between commensals and the immune system. The ability to adhere to host epithelial cells and mucus has long been considered a key property underlying colonization by both pathogenic and beneficial bacteria (34–36). Hence, under certain conditions, a host may favor the colonization and competitiveness of beneficial microbial strains, as well as can promote the secretion of specific factors able to reduce adhesion and thus, limiting a strain's colonization (34). For instance, the colonic mucus layer allows microbial adhesion and growth by exposing mucins glycans which serve as both a nutritional source and attachment sites for bacterial adhesins (37, 38). Since mucin glycosylation is distinctive for each species, this phenomenon likely favors the microbial colonization in a selective manner (39, 40). In this regard, several studies showed that altered glycosylation profiles of mucins are correlated with increased inflammation in both mouse models and humans, underlying the role of mucin glycosylation in maintenance of intestinal homeostasis (41–43). Furthermore, mucin proteins provide a nutrient harvest for intestinal microbiota. Many intestinal bacteria are known to be mucin-degrading microorganisms, involving, for instance, *Akkermansia muciniphila* (44), *Bacteroides thetaiotaomicron* (45), and *Bacteroides fragilis* (46). Released saccharides can then be used by the same bacteria as carbohydrate sources or by other resident commensals which are biologically unable to degrade polysaccharides constituting mucins (33, 47). Importantly, the mucin glycans can control microbial translocation by regulating bacterial mucin degradation.

The dense layer of transmembrane mucins that covers the entire intestinal mucosa (the mucin barrier) does not simply act as a diffusion barrier but exerts important dynamic functions that are able also to improve the intestinal immune properties, thereby regulating the interaction between commensals and the gut immune system. For instance, MUC2 is important for the cooperation between goblet cells and CD103^+^ dendritic cells that leads to T_reg_ cell differentiation and immune tolerance (48). In addition, a recent study showed that glycans associated with MUC2 induced the assembly of galectin-3-Dectin-1-FcγRIIB receptor complex and the activation of β-catenin which in turn, by inhibiting the nuclear factor-κB gene transcription, suppressed inflammatory but not tolerogenic intestinal DCs (49). Besides MUC2, MUC1 also have a strong anti-inflammatory function acting as a decoy molecule on the apical cell surface of enterocytes to limit bacterial adherence, translocation and inflammation.

Another important intestinal barrier is represented by the IEB. This anatomical structure is mainly composed of a tight junction (TJ), a adherent junction (AJ) and desmosomes. This organization is fundamental in order to maintain its integrity as well as to regulate paracellular trafficking of solutes and fluids. While AJs are necessary for assembly of TJs, the latter are made up of several kind of proteins which include transmembrane proteins (claudin, occludin), peripheral membrane proteins (zonula occludens) and regulatory molecules (2). Overall, these proteins allow the stabilization of TJs and contribute to the regulation of molecular signaling across IECs and TJs, thereby regulating gut permeability (2, 50, 51). The passage of components from the lumen through the mucosal layer is achieved by two different mechanisms, which involve paracellular and transcellular routes. The first pathway allows the passage of water, solutes and ions, and is mainly due to TJs. The regulation of paracellular permeability involve cytoskeletal contraction, endocytosis of TJ proteins, transcriptional regulation of TJ genes and epithelial cell apoptosis (52). In contrast, macromolecules such as proteins, bacterial products or microorganisms use the transcellular route which is mainly ascribed to M-cells overlying isolated lymphoid follicles or Peyer's patches (PP) (52–56).

An impairment of intestinal epithelial barrier has been recognized as a crucial mechanism for the onset of several inflammatory and immune-mediate disorders. Interestingly, increased permeability of the intestinal barrier has been ranked among those factors that may be responsible for bacterial translocation (57). Indeed, as result of gut barrier imbalances, some microorganisms, bacterial products, or toxins may translocate across the epithelium in an uncontrolled manner, leading to the onset of inflammatory disorders which may arise both in the gut and in distal organs, as described by the recent literature (50, 58–62). Current evidences have identified two main routes through which bacterial translocation may occur: transcellular or paracellular routes. While the first pathway is under the control of membrane pumps and channel, the second is due to an impairment of TJs (63). Although only a few evidences have been reported, transcellular migration has been observed in rats, where *E.coli* and *P.mirabilis* have been detected within intact enterocytes (64). Since appear to be related to an impairment of TJs, paracellular translocation has been instead associated to direct damage to enterocytes and their support structures, along with significant changes in intestinal TJs gene expression, and downregulation of both zonulin 1 and occludin (65, 66). Hence, functions of intestinal mucosa depend, at least in part, on many structural and dynamic factors, including IECs, the integrity of cellular plasma membranes, TJs and their protein components which contribute to maintain a homeostatic intestinal environment that impacts the overall health of the host.

Interestingly, the crosstalk between IECs and the mucosal immune system plays a key role to ensure the correct functioning of the immunological network operating at the intestinal level. Indeed, by detecting antigens or secreting anti-microbial molecules in response to commensal bacteria, IECs provide immune signals to these cells, thereby controlling the mucosal immune response (67).For instance, IECs sensing of gut microbial components through toll-like receptors (TRLs) protects from epithelial damage following administration of dextran sulfate sodium (DSS) (68). At the same time, intestinal immune cells exert a control on IEC-derived cytokines, such as thymic stromal lymphopoietin (TSLP), by secreting IL-12 and thus, ensuring the host ability to mount appropriate immune response toward pathogens (69). Commensal microorganisms also contribute to maintain the IEB integrity, for instance, by regulating tight junction expression and IECs proliferation (70, 71). Importantly, IECs form a real network with components of the mucosal immune system such as intraepithelial lymphocytes and immune cells residing in the intestinal lamina propria, contributing also in shaping the regulatory features of the latter in order to ensure the homeostasis. In this context, by using a culture of human monocyte-derived DCs with epithelial cells, a recent study highlights the capacity of IECs to induce anti-inflammatory DCs (72). Investigating about this phenomenon, researchers showed how this process was mediated by a combination of TSLP and other factors expressed by epithelial cells. TSLP induced-DCs did not release interleukin 12 as well as they did not drive Th1 responses toward bacteria, demonstrating that in steady state conditions the interplay between DCs and IECs is instrumental in order to maintain an anti-inflammatory environment (72).

In the intestinal barriers, gut bacteria induce also the production of secretory IgA and anti-microbial peptides (AMPs), such as RegIIIγ and alpha-defensins, which have been shown to have a crucial role in maintaining spatial compartmentalization of commensal bacteria outside the host epithelium as well as in exerting modulatory functions on both chemotaxis and TRLs signaling (73, 74). IgA also contribute to the compartmentalization of commensals by binding them and preventing their interaction with epithelial cells and their subsequent internalization. Interestingly, it has been observed that in a steady state, non- invasive bacteria residing in mice gut are coated with IgA and thus, favoring microbes to reach PP in order to induce a broader and more diversified amount of species-specific IgA in mice (22). Thus, immunoglobulin A also play a pivotal role in maintaining intestinal homeostasis.

At mucosal surfaces level, IECs-associated inflammasomes also have been demonstrated as one of those mechanisms of regulation, necessary to provide tissue homeostasis and host defense to infection (75). Several studies have described that inflammasomes play a key role in shaping the intestinal bacteria composition, such as that inflammasome-deficient mice show an aberrant microbiota, with an overgrowth of the bacterial *phyla* Bacteroidetes, particularly *Prevotellaceae* and *TM7*, and a decrease in the representation of *Lactobacillaceae* (76, 77). The subsequent dysbiosis enhances inflammatory responses in the intestine, thereby predisposing the host to develop inflammatory bowel disease (76). Moreover, mice lacking inflammasomes exhibit an imbalance in intestinal barrier functions that affect tissue homeostasis at distal organs (77, 78). Interestingly, studies in both humans and mice have identified the expression of NLRP6 inflammasome to be involved in colonic homeostasis, where it acts at the host-microbiome interface (76, 79). Specifically, it has been observed that *Nlrp6*^−/−^ mice present alterations in globet cell mucus secretion, suggesting its putative role in the maintenance of mucus barrier (80). In addition, by triggering the production of epithelial IL-18 in response to commensals, it also promotes the release of AMPs (79). Therefore, dysfunctions in this regulatory system may result in imbalances of AMPs production, thereby leading to dysbiosis and intestinal auto-immune phenomena. This has been clearly demonstrated in NLRP6 deficient mice, in which colonic epithelial cells showed reduced levels of IL-18 and altered microbiota composition (76). Interestingly, a recent study demonstrated that microbiota-associated metabolites such as taurine, histamine and spermine can modulate NLRP6 inflammasome signaling, IL-18 secretion and release of AMPs, thereby promoting intestinal dysbiosis (81). Hence, by playing a pivotal role in both intestinal mucus secretion and induction of AMPs, NLRP6 inflammasome is indirectly involve in controlling bacterial adherence and colonization.

Recently, another important gut barrier has been identified, which also play a key role in modulating the interaction between the gut bacteria and the host: the GVB. The GVB is characterized by the presence of tight junctions and adherent junctions, in addition to several cell type such as endothelial cells but also pericytes and fibroblast which are involved in the maintenance of the endothelial wall integrity (82). GVB integrity is fundamental to prevent the entry of bacteria components into the gut mucosa and systemic circulation, where they can potentially trigger activation of immune cells including self-reactive T cells. The latter hypothesis is supported by the observation that damage of the GVB is present in patients affected by an extra-intestinal autoimmune disease ankylosing spondylitis (83).

**Figure 1:**
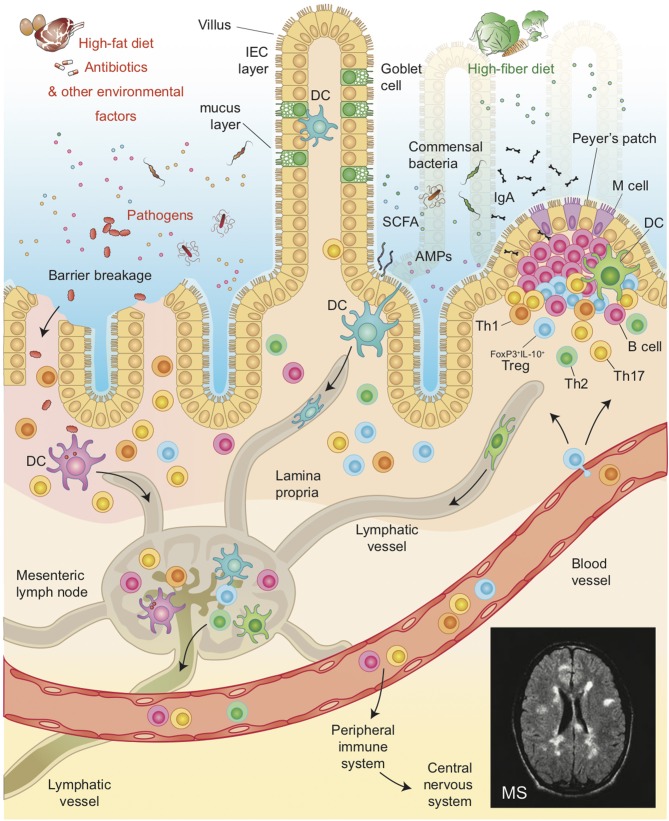
Illustration of the host intestinal barrier, including the complex interaction among IEB, and mucus layer and its role in modulating the cross-talk between commensal bacteria and the GALT. Environmental factors such as diet, pathogens or antibiotics affect the pathogenesis of MS by altering gut microbiota composition, intestinal permeability and by favoring the translocation of bacteria or microbial products, thereby shaping auto-immune responses both in the gut and in peripheral organs such as the Central Nervous System in Multiple Sclerosis.

In summary, it is now clear that the intestinal barriers play a central role in the biological network linking the intestinal microbiota and mucosal immune system, not only because it is physically and biologically positioned between them, but also because it participates actively in maintaining immune homeostasis in the gut. Hence, an imbalance of intestinal barrier or inflammation phenomena compromising its integrity, which might lead to the translocation of commensal or pathogenic bacteria, resulting in inflammation and activation of effector immune cells in the gut mucosa and, possibly, systemically and at sites distal from the intestine (Figure 1).

## How Does the Gut Environment Modulate Brain Autoimmunity?

The intestinal environment can regulate the pathogenesis of extra-intestinal autoimmune diseases such as MS through different mechanisms (84). A pro-inflammatory gut microbiota profile and alterations of the immune-regulatory function of the gut barriers could drive immune cells residing in the intestinal mucosa toward an effector phenotype. An increased differentiation of T effector cells and reduction of tolerogenic mechanisms, i.e., differentiation of T_reg_ cells (FoxP3^+^ and IL-10-secreting) may affect the functional phenotype and enhance aggressiveness T cells that circulate in the gut mucosa possibly including autoimmune myelin-reactive T cells (84). Alternatively, a damage of the different physical and biological gut barriers could favor the uncontrolled translocation of bacterial components, e.g., bacterial antigens, metabolites, toxins, from the gut mucosa into the systemic circulation where they can activate myelin-reactive T cells either in the peripheral lymphoid organs (lymph nodes) or in the brain tissue (83).

## The Role of the Microbiota

The influence of the gut microbiota in immune-mediated diseases extends beyond the intestine. Although the underlying mechanisms are yet to be characterized, several recent reports demonstrated that the gut microbiota is implied in the pathogenesis of different extra-intestinal autoimmune diseases such as Type 1 Diabetes (T1D), Rheumatoid Arthritis (RA) and MS. In support of a role of dysbiosis in the autoimmune pathogenesis of MS, there are epidemiological observations showing that many environmental factors involved in MS pathogenesis such as diet composition, hygienic conditions and the use of antibiotics, act by modifying the gut microbiota (85). In animal models of relapsing-remitting (RR) MS, it was shown that the TCR transgenic SJL/J mouse model that spontaneously develop experimental autoimmune encephalomyelitis (EAE) within 3–8 months of age, is protected from brain autoimmunity in the absence of microbiota when hosted under specific pathogen-free (SPF) conditions (84).

Importantly, alterations in the composition of the gut microbiota have also been found in individuals with RRMS compared to healthy controls. For instance, recent lines of evidence revealed overall decreased levels in both Bacteroidetes and Firmicutes *phyla* as well as in *Prevotella* strains in patients with RRMS compared to controls (86, 87). Interestingly, the alterations of gut microbiota composition, i.e., decreased relative abundance of *Prevotella*, are selectively found in RRMS patients with an active disease, meaning that those modifications could be linked with activation of brain autoimmunity and onset of MS relapse (1). Also, it is interesting to note that RRMS patients treated with Interferon-β and glatiramer acetate, show an increase in *Prevotella* genus, notoriously associated with high-fiber ingestion and beneficial role via butyrate synthesis (88). By evaluating individuals with MS, others observed a reduction in Firmicutes and *Butyricimonas*, which are butyrate-producing microorganisms (89). Likewise, another study detected an altered microbiota with decreased of bacteria with tolerogenic properties such as Clostridia XIVa and IV groups and Bacteroidetes members (87). In another study they have been found significant differences within the RR-MS patient cohort and particularly a trend toward reduced species richness in patients compared to controls (90, 91).

The mechanisms through which dysbiosis promotes MS pathogenesis are still unknown. The current hypotheses to link the gut dysbiosis with extra-intestinal autoimmune diseases such as MS hold that self-reactive T cells could be activated by mechanisms of molecular mimicry, bystander activation, and/or amplification of autoimmunity by the microbiota-induced pro-inflammatory milieu (58, 59, 92–96). For example, in animal models it has been clearly demonstrated that the gut microbiota modulate the Th17/T_reg_ balance during the development of EAE. Those experiments allowed the characterization of some commensal bacterial species able to modulate brain autoimmunity. For instance, the colonization of GF mice with SFB, selectively promotes the differentiation of Th17 lymphocytes in both the lamina propria and CNS thus worsening disease activity in EAE (84). Conversely, *Bacteroidetes fragilis* was able to prevent intestinal inflammation and brain autoimmunity through a polysaccharide A- mediated mechanisms, which induced an increase of both Foxp3^+^ T regulatory cell and IL-10-secreting Type 1 regulatory T cells (Tr1), thus limiting Th17 cell expansion (97, 98). Similarly, oral administration of *Lactobacillus* spp. and *Bifidobacterium bifidum* strongly ameliorated the EAE clinical score by increasing T_reg_ cell frequency in mice (99). Furthermore, the treatment with human gut-derived commensal *Prevotella* suppressed EAE, by decreasing pro-inflammatory Th1 and Th17 cells and enhancing T_reg_ cells frequency (100). A recent study also confirmed that in humans the gut microbiota could modulate MS pathogenesis by promoting Th17 cell differentiation in the gut. Specifically, a reduced abundance of *Prevotella* strains and increased in *Streptococcus oralis* in patients with active RRMS correlated with increased frequency of intestinal Th17 cells (1). Collectively, these research findings demonstrate that altered profiles in the microbiota composition may be directly responsible for brain autoimmunity. However, as previously mentioned, the metabolic profile induced by microbiota rather than the single bacterial strain is fundamental in shaping gut immunity and plays a role in regulating extra-intestinal autoimmune disorders and EAE. In line with this idea, mice lacking the Aryl-hydrocarbon Receptor (AhR), an environmental sensor that detects dietary, microbial and metabolic cues such as tryptophan derivatives, show an increased susceptibility to EAE and are more prone to develop infection with *Citrobacter rodentium* and *Listeria monocytogenes*. The latter finding suggests that the metabolic environment and, specifically, tryptophan derivatives metabolites, play an important role in modulating brain autoimmunity (29, 101).

## The Role of the Gut Barrier Integrity

As previously mentioned, components of the gut microbial strains could directly trigger activation of autoimmune T cells and/or their acquisition of an effector Th17 cell phenotype. However, an intestinal dysbiosis with decreased microbial function and diversity could promote extra-intestinal autoimmune diseases also by inducing inflammation and alterations of the gut barrier integrity (102, 103). In support to the latter hypothesis, studies in humans and animal models indicate that, along with intestinal inflammation and dysbiosis, an increased intestinal permeability may play a pathogenic role in brain autoimmunity (59, 95). An intestinal barrier dysfunction has been observed at the onset of EAE, in which increased permeability and altered expression of TJs have been associated to a disruption of the mucosal balance between Th1/Th17 and T_reg_ cell subsets in intestinal lamina propria, Peyer's patches and mesenteric lymph nodes (MLN) (104). A recent study furthermore showed that the degree of intestinal permeability impairment is closely related to EAE severity (105).

Also, in humans altered gut barrier functions and composition have been observed in MS patients. The intestinal barrier permeability in patients can be inferred by using a test that directly measures the ability of lactulose and mannitol to permeate the intestinal mucosa, then revealing the degree of gut permeability and mucosal alterations of the small intestine (95). A recent study, by applying this test, detected a significant increase in intestinal permeability in RRSM patients compared to healthy controls. In fact, the concentration of mannitol recovered in urine samples was strongly decreased in MS patients than in controls, suggesting the presence of abnormalities in the mechanism of intestinal absorption (95). A previous report, by using the lactulose/mannitol test, reported an increased intestinal permeability in individuals affected by RRMS and showed that the bowel inflammatory scenario correlated with systemic immune alterations (expression of the CD45RO isoform of the leukocyte common antigen on peripheral blood CD20^+^ B cells) (106). Further confirmation of diminished intestinal barrier function in MS has been provided by the detection of elevated serum zonulin levels in both RRMS and Secondary Progressive Multiple Sclerosis patients (SPMS) (107). Interestingly, in this study RRMS patients in remission phase showed serum zonulin levels comparable to controls, suggesting that an impairment of gut barrier functions may be ranked not only among those factors involved in the onset of auto-immune response, but also among those implicated in the relapse phases (107). There is another finding showing that alterations of the gut barrier integrity may lead to MS pathogenesis, namely that elevated systemic lipopolysaccharide (LPS) levels, a biomarker of increased bacterial translocation, are detectable in the plasma samples of MS individuals (68). Interesting, in those patients the bacterial translocation was associated with the presence of increased *in vitro* Th17-like responses and higher neurological disabilities (68).

## Concluding Remarks

Collectively, several lines of evidence indicate that functional and morphological alterations of the intestinal barrier are associated with dysbiosis and are present in humans and animal models of RRMS (95, 108). Persistent dysbiosis, responsible for the overgrowth of harmful bacterial species, and chronic exposures to molecules originating from microbial translocation, may shape the autoimmune T cell repertoire in MS patients toward an effector Th17 cell type interfering with T_reg_ function (109). However, although some preliminary indications exist suggesting that gut barriers are altered in MS and EAE, the putative role of the different physical and biological gut barrier in the pathogenesis of brain autoimmunity requires yet to be fully investigated.

## Author Contributions

MA reviewed the literature and wrote the manuscript. ML reviewed the literature and the final manuscript. CS reviewed the manuscript and prepared the figure. MF wrote the manuscript.

### Conflict of Interest Statement

The authors declare that the research was conducted in the absence of any commercial or financial relationships that could be construed as a potential conflict of interest.
